# Three-dimensional navigated single-position lateral lumbar interbody fusion for severely collapsed disc space with lateral osteophytes

**DOI:** 10.3171/2026.4.FOCVID2636

**Published:** 2026-07-01

**Authors:** Paul Park, Barrett Schwartz

**Affiliations:** Department of Neurosurgery, University of Tennessee Health Science Center, Semmes Murphey, Memphis, Tennessee

**Keywords:** minimally invasive, LLIF, navigation, transpsoas, single position

## Abstract

Single-position lateral decubitus lumbar interbody fusion has been shown to be an effective alternative to the classic two-stage approach. The application of 3D navigation can facilitate single-position surgery with the benefit of significantly reducing radiation exposure. In this operative video, use of 3D navigation for lateral percutaneous screw placement is highlighted, followed by key steps in leveraging navigation for the lateral lumbar interbody fusion. Nuances in the use of 3D navigation for the lateral portion of the surgery are emphasized for challenging cases with disc space collapse and lateral osteophytes.

The video can be found here: https://stream.cadmore.media/r10.3171/2026.4.FOCVID2636

## Transcript

This technique video will highlight the nuances of a navigated single-position lateral lumbar interbody fusion.

### 0:28 Presentation.

The patient is an 83-year-old gentleman with a long history of mechanical back pain with a radicular component. He has failed conservative measures. He has a normal examination.

### 0:38 X-Rays.

X-rays show multilevel spondylosis but more prominently a lytic spondylolisthesis at L3–4.

### 0:45 MRI.

MRI shows severe spondylosis with loss of disc height, grade 1 spondylolisthesis at L3–4 with high-grade foraminal stenosis.

### 0:54 Diagnosis.

There is spondylosis present at each lumbar spinal level. Stenosis, however, is only prominent at L3–4. Based on presenting symptoms and imaging findings, the L3–4 spondylolisthesis with severe foraminal stenosis was believed to be the most likely cause of his more acute symptoms. Selective treatment of L3–4 was offered given his age and comorbidities with the understanding that surgery may not address all symptoms.

### 1:14 Treatment Recommendation.

Surgery recommended was L3–4 lateral lumbar interbody fusion with posterior instrumentation in the lateral decubitus position. Three-dimensional navigation will be used to assist with not only the lateral percutaneous screw placement but also to address the lateral osteophytes, restoration of disc height, and cage placement. This will highlight application of navigation beyond screw placement.

### 1:39 Positioning.

The patient is turned to a lateral decubitus position with the left side up. A Jackson open-type frame is used with a special lateral access bed positioner, which can break the table to open the space between the lower rib and iliac crest, facilitating a transpsoas approach. Anterior view and posterior view after the table is broken.

### 2:04 Frame Placement and Navigation Registration.

An iliac pin is placed in the posterior superior aspect of iliac crest with subsequent navigation frame placement. Intraoperative image acquisition is obtained for navigation.

### 2:17 Lateral Screw Placement.

A navigated drill is utilized to determine entry sites for screw placement in the lateral decubitus position based on navigation trajectory views. After drilling a pilot hole with a navigated drill, a navigated tap is utilized to traverse the pedicle. An appropriately sized pedicle screw is connected to a screw extender and inserted into the pedicle screw track with a navigated screwdriver. After inserting the upside screws, the downside screws are then inserted in the same manner.

### 3:01 Lateral Retroperitoneal Exposure Guided by Navigation.

After screw insertion, we transition to the lateral flank where navigation is utilized to determine an entry site. Note that fluoroscopy has not been utilized. An approximately one-and-a-quarter-inch incision is made, and subcutaneous tissue is dissected until the abdominal musculature is identified. Once the abdominal musculature has been identified, the muscle-splitting approach is performed with a Kocher. After entry into the retroperitoneum with the Kocher, finger dissection is performed to create a corridor in the retroperitoneal space. A narrow-bladed retractor is then inserted and retracted peritoneum anteriorly, so that a navigated dilator with nerve stimulation can be utilized to traverse the retroperitoneal space.

### 4:00 Navigated Dilator Anchoring Into the Disc Space.

As shown in the trajectory views, the navigated dilator with nerve stimulation is advanced through the psoas muscle into the midpoint of the disc space. In this case, it is very difficult to advance the dilator into the disc space given the severity of the disc space collapse and lateral osteophytes. In these cases where the disc space is severely collapsed, the navigated dilator can be positioned in the correct trajectory, and a hammer can be utilized to advance the dilator into the disc space to be anchored.

### 4:44 Sequential Dilation and Retractor Placement.

Once the initial dilator is anchored in the disc space, sequential dilation is performed with continued nerve stimulation to minimize nerve injury. Expandable tubular retractor is placed over the final dilator and fixated in place with a table-mounted arm.

### 5:25 Exposure of the Lateral Disc.

The dilators are removed after the tubular retractor is opened, with the initial dilator left in place to mark the midpoint of the disc space. The residual psoas muscle is retracted with a narrow-bladed self-retaining retractor. There is a lateral osteophyte present that needs to be addressed to allow entry into the disc space. A navigated osteotome will be utilized to make anterior, posterior, cranial, caudal cuts in the osteophyte so it can be removed. Note, we have not used fluoroscopy as of yet due to our experience with navigation; however, with new use of navigation or with early experience, we would recommend use of periodic fluoroscopy and checking of navigation accuracy throughout the procedure. Once the bony cuts have been made with the navigated osteotome, a pituitary rongeur is utilized to remove the lateral osteophyte.

### 6:46 Disc Space Distraction and Discectomy.

The disc space is now exposed. A navigated mini-Cobb is utilized to enter the collapsed disc space. A mini-Cobb is utilized initially due to disc space collapse, so that any endplate damage if navigation is inaccurate is minimized. Haptic feedback will allow determination whether the mini-Cobb is in the disc space. This will help to determine navigation accuracy. Once navigation accuracy is confirmed, a larger navigated Cobb is utilized to obtain contralateral release. A navigated angled Cobb is then utilized to broaden the disc space release. After contralateral release with a navigated Cobb, sequential navigated distractors measuring 4 mm, 6 mm, 8 mm in height are inserted sequentially to facilitate increased disc space distraction. After sequential distraction, the disc height is substantially increased. An increased disc height allows discectomy and endplate preparation, which was performed but not shown.

### 8:18 Navigated Cage Trials.

Navigated trials are then inserted to determine length, width, and height. Since disc space height is not tracked on navigation, determination of the appropriate height is based on haptic feedback, fluoroscopic visualization, and experience.

### 8:39 Navigated Cage Insertion.

An appropriately sized cage filled with biologic is now inserted with a navigated cage inserter. Fluoroscopic picture is obtained showing appropriate placement of the cage with increased disc height restoration and reduction in spondylolisthesis.

### 9:25 Rod Placement.

The bed is now tilted anteriorly, and pre-cut and contoured rods are inserted through the screw heads followed by placement of the set screws. Final x-rays obtained showing good height restoration with reduction of spondylolisthesis with appropriate hardware placement.

### 9:44 Closure and Postoperative Course.

Estimated blood loss was 20 mL, and the patient was discharged home on postoperative day 1.

### 9:49 Discussion.

The single-position lateral lumbar interbody fusion is an effective alternative to the classic two-stage approach.^1^ Single-position surgery reduces operative time compared to repositioning between the posterior and lateral approaches.^2,3^ Three-dimensional navigation significantly reduces radiation exposure and maintains high accuracy for instrumentation and cage placement.^4,5^ This video provides a step-by-step technique for incorporating 3D navigation into every aspect of the single-position lateral lumbar interbody fusion with posterior instrumentation.

## Disclosures

P. Park reported other royalties from Globus; personal fees from Globus, Medtronic, Cerapedics, and Kuros; stock in ATEC; and grants from Cerapedics, ISSG, and SMISS outside the submitted work.

## Author Contributions

Primary surgeon: Park. Assistant surgeon: Schwartz. Editing and drafting the video and abstract: Park. Critically revising the work: Park. Reviewed submitted version of the work: Park. Approved the final version of the work on behalf of both authors: Park. Supervision: Park.

## Supplemental Information

### Patient Informed Consent

The necessary patient informed consent was obtained in this study.
